# Cross-species and cross-border spread of *aph(3′)-ia*-mediated neomycin resistance in *Escherichia coli* from animals: a genomic perspective

**DOI:** 10.1093/jac/dkag143

**Published:** 2026-04-17

**Authors:** Mattia Pirolo, Manal Abuoun, Nicholas Duggett, Marisa Haenni, Mikaela K Fritz, Oskar Nilsson, Kees T Veldman, Michael S M Brouwer, Jade Davies, Emma Stubberfield, Peter Damborg, Luca Guardabassi

**Affiliations:** Department of Veterinary and Animal Sciences, University of Copenhagen, Frederiksberg C, Denmark; Department of Bacteriology, Animal and Plant Health Agency, Weybridge, Surrey, UK; Department of Bacteriology, Animal and Plant Health Agency, Weybridge, Surrey, UK; ANSES—Université de Lyon, Unité Antibiorésistance et Virulence Bactériennes, Lyon, France; Department of Animal Health and Antimicrobial Strategies, Swedish Veterinary Agency, Uppsala, Sweden; Department of Animal Health and Antimicrobial Strategies, Swedish Veterinary Agency, Uppsala, Sweden; Department of Bacteriology, Host Pathogen Interaction & Diagnostics, Wageningen Bioveterinary Research, Lelystad, the Netherlands; Department of Bacteriology, Host Pathogen Interaction & Diagnostics, Wageningen Bioveterinary Research, Lelystad, the Netherlands; Department of Bacteriology, Animal and Plant Health Agency, Weybridge, Surrey, UK; Department of Bacteriology, Animal and Plant Health Agency, Weybridge, Surrey, UK; Department of Veterinary and Animal Sciences, University of Copenhagen, Frederiksberg C, Denmark; Department of Veterinary and Animal Sciences, University of Copenhagen, Frederiksberg C, Denmark

## Abstract

**Background and objectives:**

Neomycin is widely used in livestock to control colibacillosis. Resistance among clinical *Escherichia coli* is increasing, likely driven by the use of this aminoglycoside following the European restrictions on zinc oxide and colistin. The aim of this study was to investigate the genetic epidemiology of neomycin-resistant *E. coli* across six animal species and five European countries.

**Methods:**

A total of 750 neomycin-resistant *E. coli* isolates were included, combining retrospectively identified genomes with prospectively isolated and sequenced strains from multiple animal species. Genomic analyses, based on short reads (*n* = 529), long reads (*n* = 177), or hybrid assemblies (*n* = 44), included phylogenetic comparison, and identification of neomycin resistance genes and their genetic contexts.

**Results:**

Neomycin resistance was mediated by *aph(3′)-Ia* in 98.2% of the isolates, which were distributed across 28 clonal complexes, predominantly CC10, which was frequently detected in all countries and hosts. *aph(3′)-Ia* was primarily carried by conjugative plasmids of diverse replicon types (70.3%) and embedded in two transposons (Tn*903* and Tn*4352*) and three newly identified genetic elements, while a chromosomally integrated element accounted for the remaining 29.7%. Plasmids carrying *aph(3′)-Ia* frequently co-harboured tetracycline and trimethoprim-sulfamethoxazole resistance genes, with IncX1 plasmids additionally containing chloramphenicol and quinolone resistance determinants. Phylogenetic analysis revealed dissemination of plasmid-borne elements across multiple lineages, while the chromosomal element was mostly restricted to ST117, ST88, and ST189.

**Conclusions:**

The spread of neomycin resistance in *E. coli* from European livestock is primarily driven by the mobility of *aph(3′)-Ia* embedded in diverse genetic contexts and plasmid backbones, promoting co-transfer of other resistance determinants.

## Introduction

*Escherichia coli* is one of the most common Gram-negative opportunistic bacterial pathogens in animals and humans, causing a wide variety of gastrointestinal and extra-intestinal diseases, such as enteritis, sepsis, and urinary tract infections. Animal-adapted *E. coli* pathotypes, such as enterotoxigenic (ETEC) and avian pathogenic (APEC) strains, are responsible for colibacillosis, including neonatal and post-weaning diarrhoea in pigs, diarrhoea in newborn calves and various systemic infections in poultry.^1–3^ At the same time, *E. coli* is also a common commensal species inhabiting the intestinal tract of all animal species, including humans, and can act as a reservoir for exchange of antimicrobial resistance genes and mobile genetic elements across hosts.

Neomycin is an aminoglycoside antibiotic approved in Europe for treatment and control of colibacillosis in cattle, pigs, sheep, goats and poultry by the oral route and to treat bovine mastitis by intramammary administration. Due to its poor gastrointestinal absorption, orally administered neomycin is primarily effective against localized intestinal infections rather than systemic infections.^4^ In *E. coli*, neomycin resistance is predominantly mediated by aminoglycoside-modifying enzymes such as aminoglycoside 3*′* O-phosphotransferases [*aph(3′)*], including *aph(3′)-Ia*, *aph(3′)-Ib*, and *aph(3′)-IIa*.^4^ These genes are mostly located on mobile genetic elements such as transposons,^5^ facilitating their horizontal movement between different clonal lineages and bacterial species.

National surveillance reports indicate a concerning rise in the prevalence of neomycin-resistant *E. coli* isolated from animal infections. In Sweden, resistance levels among clinical isolates from calves and pigs reached 11% and 12.5% in 2024, respectively, paralleling recent increases in neomycin use.^6^ In the United Kingdom, 19% of livestock isolates were neomycin-resistant in 2021, with the highest levels (30%–35%) observed in pre-weaned and neonatal calves.^7^ In the Netherlands, resistance ranged from 4%–9% in pigs and 21%–25% in cattle between 2020 and 2024.^8^ In France, neomycin resistance exceeded 50% in diarrheic calves and approached 20% in diseased pigs in 2024.^9^ The most pronounced increase has been observed in Denmark, where following restrictions on colistin and zinc oxide,^10^ neomycin use and resistance rates surged among porcine clinical isolates,^11^ reaching 60% in haemolytic *E. coli* by 2024.^12^ This rise was linked to the dissemination of *aph(3′)-Ia* on distinct plasmid backbones within two dominant lineages, namely sequence type (ST) 100 and clonal complex (CC) 10.^13^

Neomycin-resistant *E. coli* have also been reported in humans, food products, and urban sewage worldwide,^14–17^ underscoring its global and One Health relevance. Hence, we hypothesize that the dissemination of neomycin-resistant *E. coli* lineages and mobile elements extends beyond host species and geographic boundaries. In line with this hypothesis, in this study we investigated the dynamics of neomycin resistance and the associated genetic elements in *E. coli* from different animal sources and geographical locations in five European countries, namely Denmark, France, the Netherlands, Sweden and the United Kingdom. By employing a comprehensive genomic analysis, we aimed at elucidating the mechanisms driving the spread of neomycin-resistant *E. coli* by mapping the distribution of neomycin-resistant *E. coli* lineages and plasmids in Europe.

## Materials and methods

### Bacterial isolates

*Escherichia coli* isolates included in this study were identified through a combination of retrospective and prospective screening. Retrospective screening included (i) the examination of existing collections of sequenced isolates for the predominant neomycin resistance genes (e.g. *aph(3′)-Ia*, *aph(3′)-Ib* and *aph(3′)-IIa*) and (ii) the screening of stored clinical and non-clinical isolates from livestock animals using a standardized process, including culture on selective media (e.g. MacConkey agar supplemented with 8 mg/L of neomycin) and PCR-based confirmation of neomycin resistance genes.^18^ Prospective screening included (i) the screening of livestock faecal samples for neomycin-resistant *E. coli* isolates and (ii) the identification of neomycin-resistant clinical *E. coli* isolates from veterinary diagnostic laboratories participating in national surveillance programmes. Metadata for all isolates are provided in Dataset S1 (available as Supplementary data at *JAC* Online). Minimum inhibitory concentration (MIC) of neomycin was tested by broth microdilution according to EUCAST guidelines (https://www.eucast.org/bacteria/methodology-and-instructions/mic-determination/), and results were interpreted according to epidemiological cut-offs (ECOFFs) available at https://www.eucast.org/. The upper MIC ranges varied between laboratories, but all included concentrations ≥16 mg/L, corresponding to the EUCAST epidemiological cut-off (ECOFF) for non-wild-type *E. coli*. Descriptions of the isolates included from each country, as well as methods for DNA extraction and whole-genome sequencing of newly identified isolates, are detailed in the Supplementary Methods.

### Genome quality check and typing

*In silico* analysis followed the pipeline illustrated in Figure S1. After reads assembly (Supplementary Methods), genome quality was checked with QUAST and CheckM,^19,20^ and only assemblies meeting stringent inclusion criteria (completeness ≥99%, contamination ≤2%, and fewer than 1000 contigs) were included for further analysis. Genomes were screened for the presence of neomycin resistance determinants using ABRicate (https://github.com/tseemann/abricate) against the ResFinder database,^21^ and alignment results with identity and coverage scores greater than 95% were selected as positive matches. Multi-locus sequence typing (MLST) was performed using mlst (https://github.com/tseemann/mlst) with the Achtman scheme, and clonal complex (CC) clustering was performed by Phyloviz.^22^ Phylogroups were determined using ClermonTyping.^23^

### Genetic context and plasmid analysis

The *aph(3′)-Ia* genetic context was analysed in genomes where the gene was located on contigs ≥2000 bp. After extraction of these contigs, they were annotated using Prokka,^24^ and the *aph(3′)-Ia* genetic element was identified through BLAST screening and manual inspection, and representative sequences were aligned and visualized using clinker.^25^ Chromosomal or plasmid origin of the contigs was determined using RFPlasmid.^26^ Plasmids carrying *aph(3′)-Ia* were constructed with MOB-suite,^27^ which was also used to infer replicon types and mobility. Plasmids were screened for replicon types and antimicrobial resistance genes using ABRicate against the PlasmidFinder and ResFinder databases, respectively, and were visualized with BRIG.^28^ Class 1 integrons were predicted using IntegronFinder.^29^

### Phylogenetic analysis

Genomes with a defined *aph(3′)-Ia* element were annotated using Prokka,^24^ and the resulting.gff files were used to determine a core genome alignment via Roary.^30^ A maximum likelihood (GTR + G model) phylogenetic tree was constructed using IQ-TREE and visualized via iTOL.^31,32^

### Statistical analysis

Association between ST/CC and *aph(3′)-Ia* genetic elements with hosts or countries was assessed with pairwise Fisher’s exact test, with Benjamini-Hochberg correction of *P*-values for false discovery rate. Significance was set at *P* < 0.05.

### Data availability

Sequencing data from Danish isolates are available in the NCBI Sequence Read Archive under BioProjects PRJEB38608, PRJNA849907 and PRJNA934822. French data are available under BioProjects PRJEB50837 and PRJNA1226120. Dutch data are available under BioProjects PRJNA885502 and PRJEB90648. United Kingdom data are available under BioProjects PRJNA605147. Swedish data are available under BioProject PRJEB84943.

## Results

### Bacterial isolates and epidemiological data

**Figure 1.**
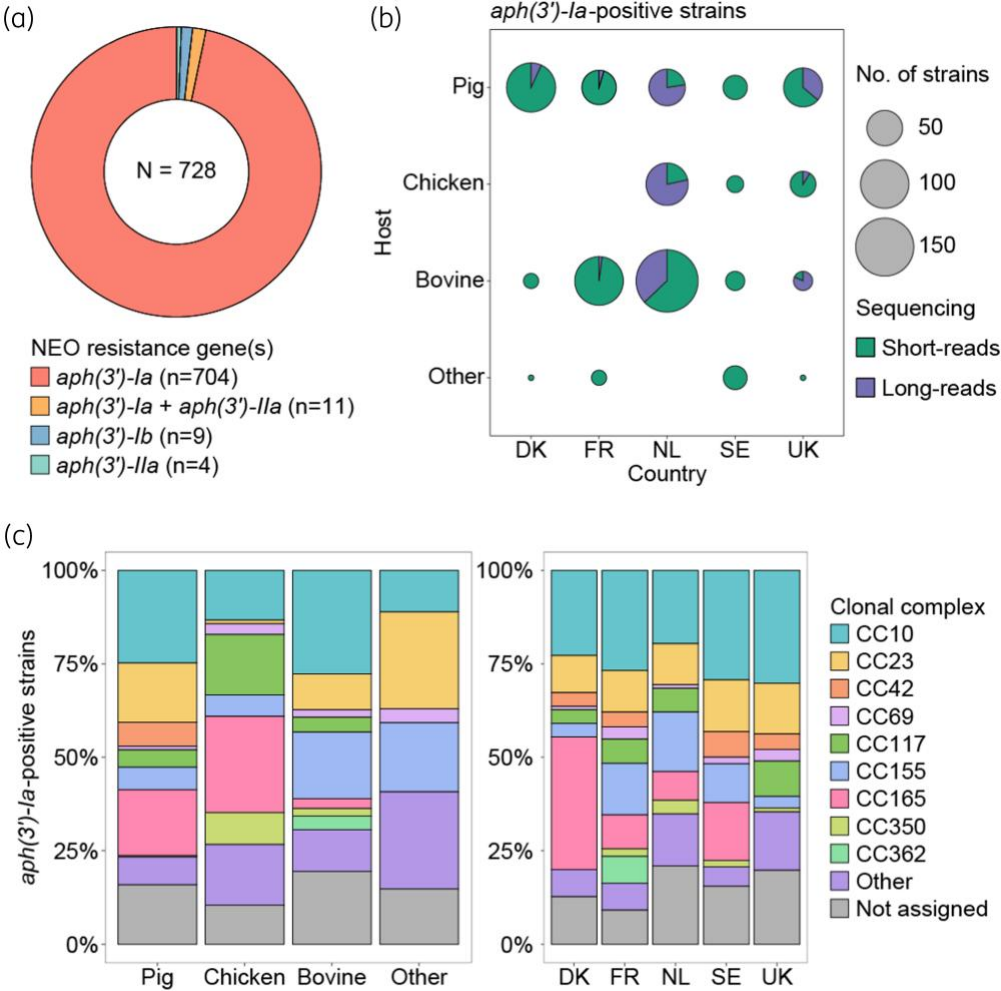
Molecular epidemiology of neomycin-resistant *E. coli* across host and countries. (a) Frequency of neomycin resistance genes across 728 *E. coli* genomes. (b) Number of *aph(3′)-Ia*-positive isolates (*n* = 715) stratified by sequencing method, host and country of origin. (c) Distribution of major clonal complexes (CCs) across hosts and countries.

After quality filtering, 750 genomes were included in the study, originating from retrospective (*n* = 494) and prospective (*n* = 256) screenings of isolates (Dataset S1). Data collection comprised 529 genomes sequenced using short-read, 177 sequenced with long-read, and 44 sequenced with both approaches. Of the included genomes, 728 (97.1%) were positive for at least one neomycin-resistance gene (Figure 1a). In the remaining 22 genomes, no genes explaining the phenotypic resistance (*i.e.* MIC ≥16 mg/L by broth microdilution) were detected.

The *aph(3′)-Ia* gene was identified in 715 isolates (98.2% of neomycin-resistant isolates). Notably, four isolates carried two copies of this gene. Fifteen (2.1%) isolates carried *aph(3′)-IIa*, either alone (*n* = 4 isolates) or in combination with *aph(3′)-Ia* (*n* = 11 isolates) (Figure 1a). Nine porcine isolates from Denmark carried *aph(3′)-Ib* (1.4%).

The *aph(3′)-Ia*-positive isolates originated from diverse sources across five countries and were grouped into four categories: pigs, poultry, cattle, and other sources (primarily companion animals and horses), with cattle (42.2%) and pigs (39.3%) representing the main hosts in this collection of isolates, although this distribution varied among countries (Figure 1b). The majority of *aph(3′)-Ia*-positive isolates were collected during or after 2015 (668/715, 93.4%) (Figure S2a). In total, 254 (35.5%) *aph(3′)-Ia*-positive isolates were isolated from clinical cases, while 261 (35.6%) genomes were obtained from national screening for extended-spectrum beta-lactamase (ESBL) in livestock farming. The year of isolation, the proportions of clinical and commensal isolates, and the distribution of ESBL in each host and country are shown in Figure S2b and c, respectively.

The *aph(3′)-Ia*-positive isolates were assigned to 155 different STs, with only six isolates (0.8%) remaining unassigned. CC assignment grouped 597 isolates (83.5%) into 28 distinct CCs. Although most STs and CCs were detected across multiple hosts and countries (Figure 1c and Figure S3), host- and country-specific associations emerged. CC10 was the most prevalent and widely distributed lineage across all countries (19.3–30.2%) and hosts (11.1–27.5%), with ST10 accounting for 62.1% (105/169) of isolates belonging to this lineage. CC10 showed a significantly higher prevalence in pigs (24.6%, *P* = 0.044) and cattle (27.8%, *P* = 0.002) compared to poultry (13.5%). Similarly, CC23 was identified in all countries (10.0–14.0%), but was significantly more frequent in pigs (16.1%, *P* < 0.001) and cattle (9.1%, *P* = 0.002) compared to poultry (1.0%). Although CC117 (ST117) and ST58 (CC155) were isolated from multiple host species, they exhibited significant associations (*P* < 0.05) with poultry and cattle, respectively. Within CC165, ST100 was found exclusively in pigs from Denmark and France, while ST189 was strongly associated with poultry (*P* < 0.05). Notably, ST1433, which was not assigned to any known CC, was exclusively recovered from Dutch cattle, predominantly among ESBL-producing isolates.

### Analysis of the aph(3*′*)-ia context

**Figure 2.**
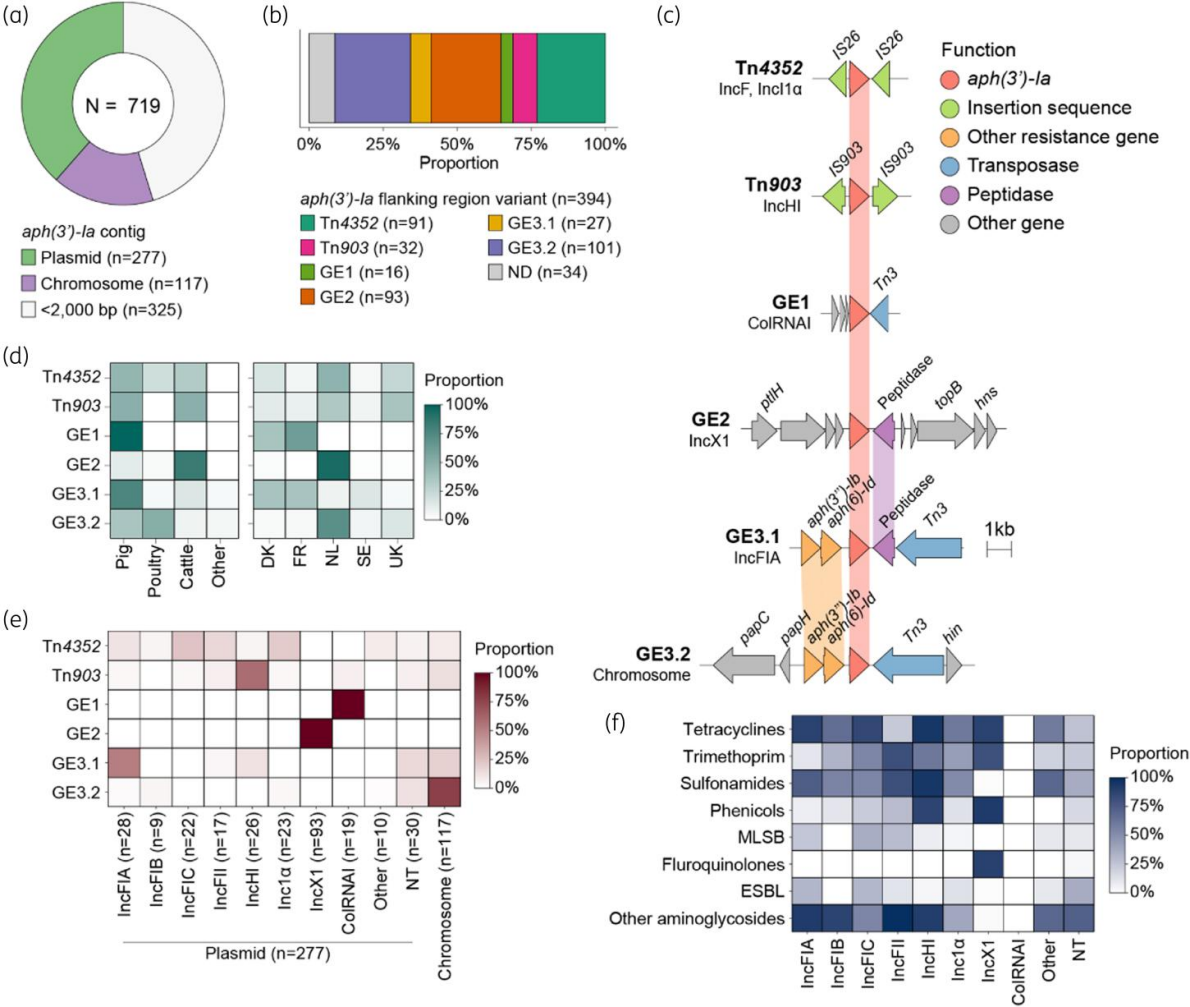
Analysis of the *aph(3′)-Ia* location and its genetic context. (a) Classification of *aph(3′)-Ia* contigs by RFPlasmid. Contigs shorter than 2000 bp (42.1%) were excluded from the analysis. (b) Proportions of six *aph(3′)-Ia* genetic elements identified on contigs ≥2000 bp (*n* = 394). (c) Alignment of representative sequences for the six *aph(3′)-Ia* genetic elements. Genes with identity ≥95% are connected with coloured lines. (d) Distribution of the six genetic elements between main hosts and countries. (e) Distribution of the six genetic elements between plasmids reconstructed with MOB-suite and chromosomal sequences. (f) Distribution of resistance determinants by antimicrobial class across plasmid replicon types. Abbreviations: ESBL, extended spectrum beta-lactamase; GE, genetic element; MLSB, macrolide-lincosamide-streptogramin B; ND, not determined; NT, not-typeable plasmid.

To investigate the genetic context of *aph(3′)-Ia*, contigs containing this gene (*n* = 719, from 715 isolates) were extracted and annotated. Contigs <2000 bp (45.2%) were excluded to ensure sufficient flanking sequence for downstream analysis. *aph(3′)-Ia* was predicted by RFPlasmid to be plasmid- and chromosome-associated in 70.3% and 29.7% of the remaining 394 contigs, respectively (Figure 2a). Genetic context analysis, combining BLAST screening and manual inspection, resolved six distinct elements, including the previously described transposons Tn*4352* (23.1%) and Tn*903* (8.1%), as well as four newly identified genetic elements (GE): GE1 (4.1%), GE2 (23.6%), GE3.1 (6.9%), and GE3.2 (25.6%) (Figure 2b). Thirty-four contigs (8.3%) could not be assigned to a genetic element due to truncations upstream or downstream of the *aph(3′)-Ia* gene. Representative alignments of the six genetic elements are shown in Figure 2c.

The six elements showed different distribution patterns among hosts and countries (Figure 2d). Both Tn*4352* and Tn*903* were detected in all countries and were broadly distributed among hosts. However, Tn*903* was notably absent in poultry isolates. GE1 was exclusively identified in pigs and was restricted to isolates from Denmark and France. Similarly, GE3.1 showed a significant association with pigs (77.8% of carriers), being more prevalent than in cattle (14.8%, *P* = 0.005) or poultry (3.7%; *P* = 0.014), and was especially prevalent in Danish and French isolates (*P* < 0.05 for all pairwise comparisons). GE2 showed a significant association with cattle (84.9% of carriers), with significantly lower prevalence in pigs (11.8%, *P* < 0.001) and poultry (3.2%, *P* < 0.001) and almost exclusively prevalent among Dutch isolates (95.7%, *P* < 0.001 in all pairwise comparisons). Lastly, GE3.2 was significantly associated with poultry (51.5% of carriers, *P* < 0.05 in pairwise comparison).

### Analysis of the aph(3*′*)-ia location

To determine the genomic localization of the six identified genetic elements, a total of 277 *aph(3′)-Ia*-containing plasmids were reconstructed from contigs using MOB-suite from short- (*n* = 143) and long-read (*n* = 134) genomes (Dataset S2). Thirteen replicon types were identified, with IncX1 (33.6%), IncFIA (10.1%), and IncI1α (8.3%) being the most common. These plasmids spanned sizes of 45 kb for IncX1 up to 220 kb for IncFIA and 130 kb for IncI1α, with representative plasmid maps shown in Figure S4. The distribution of predicted plasmid length for all replicon types is shown in Figure S5a. Replicon type was not resolved for 30 plasmids (10.8%). Plasmid mobility predictions indicated that 80.9% of *aph(3′)-Ia*-containing plasmids were conjugative, including all IncFIB, IncFIC, and IncX1 plasmids (Figure S5b), while all ColRNAI plasmids (7.6%) were predicted as mobilizable (*i.e.* containing either a relaxase or an oriT but missing the mate-pair formation markers).

A strong correlation was observed between genetic elements and plasmid replicon types or chromosomal sequences (Figure 2e). GE1 and GE2 were exclusively found in ColRNAI and IncX1 plasmids, respectively, while GE3.1 and Tn*903* were predominantly associated with IncFIA (51.9%) and IncHI (both HI1 and HI2; 59.4%) plasmids, respectively. Tn*4352* was distributed across multiple plasmid types, primarily IncF-types and Inc1α. GE3.2 was frequently found on the chromosome (78.2%). These associations were further supported by BLASTn search of representative sequences (top 100 hits, ≥95% identity and coverage; Dataset S3), which revealed consistent pairing between GE1 and ColRNAI plasmids from *E. coli* and *Salmonella enterica*, GE2 and IncX1 plasmids from *E. coli* and *Shigella flexneri*, GE3.1 and large IncHI/IncFIA plasmids, and GE3.2 with chromosomal regions of *E. coli* (Figure S6).

Except for small ColRNAI plasmids, resistance gene screening revealed high frequencies of co-localized resistance genes on all plasmids. Tetracycline [primarily *tet(A)* and *tet(B)*], trimethoprim (*dfrA*) and sulphonamide (*sul1*, *sul2*, and *sul3*) resistance genes were widely distributed across plasmid types (Figure 2f and Figure S7). Screening with IntegronFinder identified complete class 1 integrons in 49.8% of reconstructed plasmids (138/277). Among plasmids carrying *dfrA*, 62.9% (90/143) encoded *dfrA* within a class 1 integron cassette, while tetracycline and sulphonamide resistance genes were not detected within integron structures. Additionally, *floR* and *qnrS1*, conferring resistance to chloramphenicol and quinolones, respectively, were highly prevalent in IncX1 plasmids, with frequencies of 93.5% and 90.3%, respectively, within this replicon type.

### Phylogenetic analysis of aph(3*′*)-ia-carrying isolates

**Figure 3.**
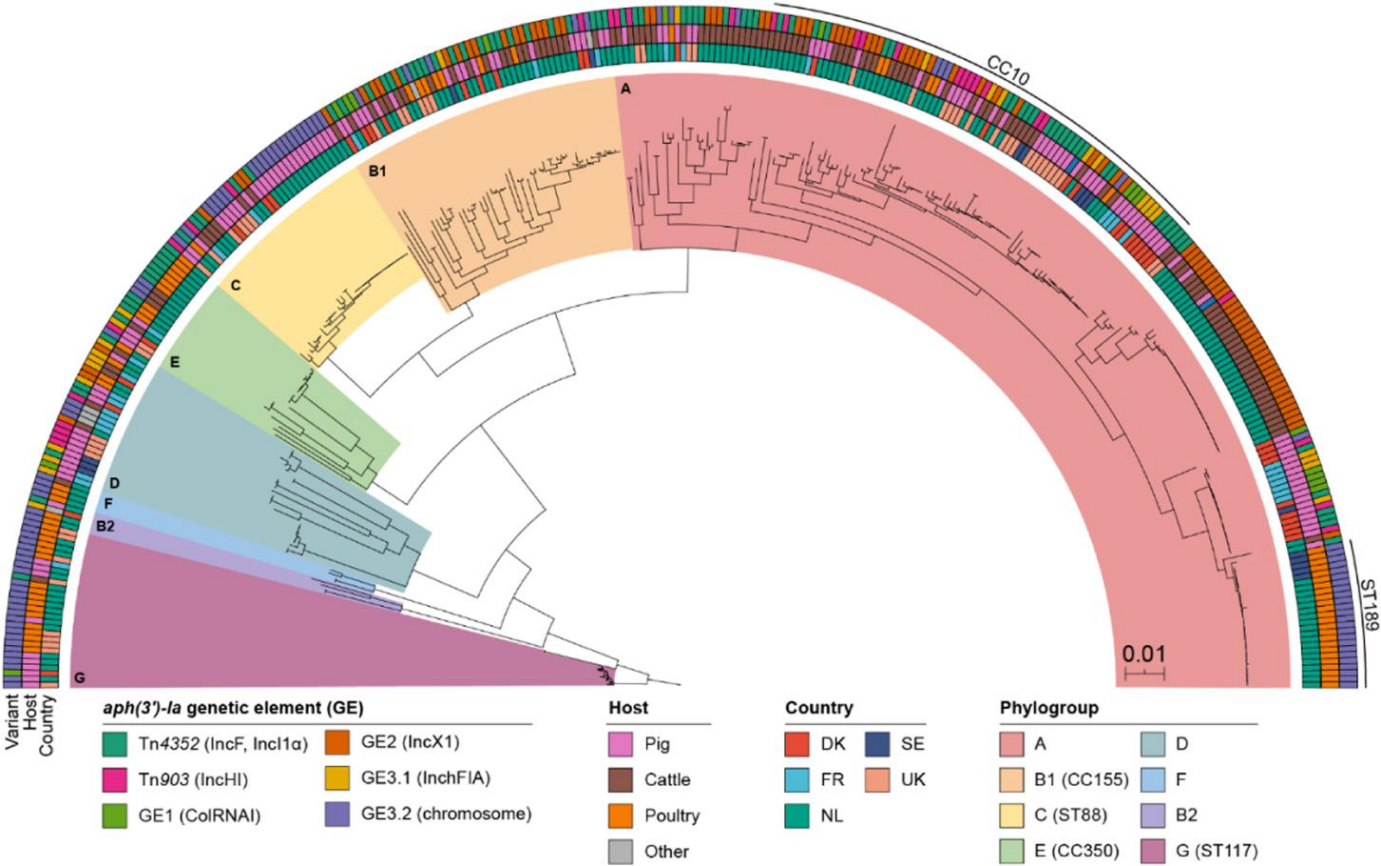
Core-gene phylogenetic analysis of 356 neomycin-resistant *E. coli* isolates for which the *aph(3′)-Ia* genetic element was defined. Maximum likelihood (GTR+G model) phylogenetic tree based on 1.75 Mbp (33.7 ± 1.9% of the genome) with the main clusters linked with phylogroups. The main sequence type (ST) or clonal complex (CC) of specific phylogroups is indicated in the phylogroup legend. The tree was arbitrarily rooted on phylogroup D isolates (*n* = 31). Metadata on isolates country of origin, host and *aph(3′)-Ia* genetic element are illustrated in the inner, middle and outer circle, respectively.

A maximum likelihood phylogenetic tree was constructed using the GTR+G model based on a core-genome alignment of 356 neomycin-resistant isolates with a defined *aph(3′)-Ia* genetic element (Figure 3). The phylogeny was predominantly composed of isolates from the Netherlands (*n* = 215, 60.4%), reflecting the high proportion of long-read sequenced genomes available from this country. Isolates from the United Kingdom, Denmark, France, and Sweden accounted for 14.9% (*n* = 53), 11.2% (*n* = 40), 8.7% (*n* = 31), and 4.8% (*n* = 17), respectively.

Eight major clusters were identified, aligning with the phylogroup assignments. Isolates from different countries and hosts were intermingled throughout the phylogeny, consistent with MLST results. Plasmid-borne *aph(3′)-Ia* genetic elements were found across the phylogeny, reflecting their broad dissemination. In contrast, the chromosomal element (GE3.2) was confined to three distinct clusters, namely phylogroup G (ST117), phylogroup C (ST88), and ST189 within phylogroup A.

## Discussion

We conducted a comprehensive investigation into the genetic epidemiology and dissemination of neomycin resistance in *E. coli* isolated from animals, primarily livestock, across five European countries. Our analysis confirmed *aph(3′)-Ia* as the main genetic determinant of neomycin resistance, present in 98.2% of isolates, regardless of hosts or country of origin.

Our analysis revealed extensive diversity in the genomic contexts carrying *aph(3′)-Ia*, encompassing five distinct plasmid backbones and one chromosomal integration. Two well-characterized composite transposons, Tn*4352* and Tn*903*, were detected among these contexts, flanked by IS*26* and IS*903*, respectively.^33,34^ While IS*26* is a recognized driver of multidrug resistance gene assembly and mobilization,^35^ the transposition activity of IS*903*-family elements remains less understood and may constitute an underexplored route for aminoglycoside resistance dissemination.

Early work had already reported *aph(3′)-Ia* on both large and small plasmids as well as within chromosomal loci,^36^ and recent phylogenomic analyses suggest *Klebsiella michiganensis* as a possible ancestral reservoir of this gene.^37^ Here, we expand this knowledge by revealing multiple independent acquisition events and novel genomic architectures. Four previously undescribed *aph(3′)-Ia* genetic elements (GE1–GE3.2) were identified. Unlike classical transposons, these elements are not flanked by insertion sequences, and may represent non-canonical derivatives or recombination products of known mobile elements.

Epidemiological analysis of *aph(3′)-Ia*-positive isolates revealed two predominant CCs, CC10 and CC23, which were widely disseminated across hosts and countries. Both CCs, primarily represented by ST10 and ST88, were particularly enriched among pigs and cattle, consistent with their frequent detection in food-producing animals and their potential role in cross-species transmission.^38^ Additional host-associated sequence types included ST100 in pigs, ST58 in cattle, and ST117 in poultry. ST58 has been increasingly reported in cattle and environmental sources worldwide,^39^ whereas ST117 is a well-recognized APEC lineage frequently implicated in extraintestinal pathogenic *E. coli* (ExPEC) infections in poultry and humans.^40,41^ The occurrence of *aph(3′)-Ia*-carrying plasmids within such epidemiologically successful lineages suggests that dissemination of neomycin resistance follows a dual mechanism driven by antimicrobial selection pressure in livestock: first, through horizontal transfer of mobile elements among cohabiting *E. coli* populations, and second, through clonal expansion of host-adapted strains that serve as stable reservoirs within animal production systems.

Despite the overall diversity of genetic context, the strong associations between certain genetic elements and specific plasmid replicon types or chromosomal integration sites point to multiple, independent acquisition events of *aph(3′)-Ia* within the *E. coli* livestock population. In the case of plasmid-borne genetic elements, these acquisitions were likely followed by horizontal dissemination across different *E. coli* lineages, facilitated by the high proportion (∼90%) of conjugative or mobilizable plasmids. For the chromosomal element, the presence of *aph(3′)-Ia* in multiple lineages (*e.g*. ST88, ST117, and ST189) suggests that once stably integrated, the gene spreads through clonal expansion, potentially driven by the selective pressure associated with increased neomycin use in livestock production.

The high prevalence of conjugative plasmids underscores their central role in horizontal gene transfer and in facilitating co-selection, whereby exposure to one antimicrobial class promotes the persistence of plasmids carrying resistance to additional drug classes. This is particularly relevant in livestock systems, where tetracyclines and trimethoprim–sulfamethoxazole are widely used,^42^ creating selective conditions favourable to the maintenance of multi-resistant plasmids. In our dataset, this was reflected by the frequent co-occurrence of *tet*, *dfrA* and *sul* genes on *aph(3′)-Ia*–carrying plasmids, most notably in IncX1 plasmids circulating among Dutch bovine isolates, which also frequently harboured chloramphenicol and quinolone resistance genes.

Beyond livestock, similar multidrug resistance *aph(3′)-Ia* plasmids have been increasingly reported in *E. coli* and *Salmonella* isolates from diverse sources, including poultry, wild animals, food products, and environmental reservoirs. IncX1 plasmids harbouring *aph(3′)-Ia* together with *qnrS1* and *tetA* have been detected in *E. coli* from poultry in Poland,^43^ whereas IncHI and IncF plasmids carrying *aph(3′)-Ia* alongside *floR*, *sul*, and *dfrA* genes have been described in *E. coli* from deer, pigs, and poultry in Asia.^44–47^ Comparable IncF and IncHI plasmid structures containing *aph(3′)-Ia* and co-located resistance genes have also been found in wastewater metagenomes,^14^ and in *Salmonella* from livestock.^47,48^ Collectively, these observations indicate that the co-localization of *aph(3′)-Ia* with genes conferring resistance to tetracyclines, sulphonamides, and chloramphenicol, as observed in our dataset, mirrors a globally disseminated resistome architecture that extends beyond host species and geographic boundaries. This convergence across animal, environmental, and foodborne isolates highlights the One Health dimension of neomycin resistance and underscores the potential for cross-sectoral dissemination of multidrug-resistant plasmids. Future studies should investigate the selective forces driving the persistence of these plasmids under different antimicrobial usage regimes and assess their potential transmission across the animal–human interface. Integrating genomic surveillance of high-risk plasmid backbones into existing antimicrobial resistance monitoring frameworks may further support evidence-based stewardship strategies aimed at mitigating co-selection and limiting the long-term spread of multidrug resistance in livestock production systems.

We acknowledge some study limitations. First, the sampling was uneven across countries, with a strong overrepresentation of Dutch isolates, particularly in the long-read dataset, which may have biased the observed distribution of *aph(3′)-Ia* genetic elements and influenced phylogenetic clustering. Second, nearly half (45.2%) of *aph(3′)-Ia*-carrying contigs were too short to allow confident reconstruction of their genetic context, potentially leading to an underestimation of the true diversity of *aph(3′)-Ia* genetic arrangements and biasing the analysis toward more prevalent or better-characterized genetic elements. Third, a substantial proportion of the dataset consisted of ESBL-producing isolates derived from national surveillance programmes (35.6%), which may have skewed the co-resistance profiles and overrepresented resistance genes unrelated to neomycin resistance. Finally, the identification of co-located resistance genes and plasmid mobility was based on *in silico* predictions from closed (*n* = 134) but also contigs inferred (*n* = 143) plasmids and was not experimentally validated through conjugation or plasmid stability assays.

In conclusion, this study provides a comprehensive genomic overview of neomycin resistance in *E. coli* from livestock across Europe, highlighting *aph(3′)-Ia* as the predominant resistance determinant. We show that this gene is embedded in diverse genetic contexts, yet it exhibits strong associations with specific plasmid replicon types or chromosomal integration, suggesting independent acquisition events followed by horizontal dissemination or clonal expansion. The frequent co-localization of *aph(3′)-Ia* with other resistance genes on conjugative plasmids raises concerns about co-selection and the spread of multidrug resistance in livestock production.

## Supplementary Material

dkag143_Supplementary_Data

## Supplementary data

Figures S1 to S7 are available as Supplementary data at *JAC* Online.
