# Supplementary material for: Cross-species and cross-border spread of aph(3′)-ia-mediated neomycin resistance in Escherichia coli from animals: a genomic perspective
Source: J Antimicrob Chemother. 2026 Apr 17;81(5):dkag143. doi: 10.1093/jac/dkag143 (PMC13089612; doi:10.1093/jac/dkag143)
Supplement: dkag143_Supplementary_Data [file dkag143_supplementary_data.zip › Supplementary Methods and Figures_R1.docx]

**Cross-species and cross-border spread of *aph(3′)-Ia*-mediated neomycin resistance in *Escherichia coli* from animals: a genomic perspective**

Mattia PIROLO^1^*, Manal ABUOUN^2^, Nicholas DUGGETT^2^, Marisa HAENNI^3^, Mikaela K. FRITZ^4^, Oskar NILSSON^4^, Kees T. VELDMAN^5^, Michael S. M. BROUWER^5^, Jade DAVIES^2^, Emma STUBBERFIELD^2^, Peter DAMBORG^1^ and Luca GUARDABASSI^1^

^1^Department of Veterinary and Animal Sciences, University of Copenhagen, Frederiksberg C, Denmark

^2^Department of Bacteriology, Animal and Plant Health Agency, Weybridge, Surrey, United Kingdom

^3^ANSES - Université de Lyon, Unité Antibiorésistance et Virulence Bactériennes, Lyon, France

^4^Department of Animal Health and Antimicrobial Strategies, Swedish Veterinary Agency, Uppsala, Sweden

^5^Wageningen Bioveterinary Research Part of Wageningen University and Research, Lelystad, the Netherlands

*Correspondence: mapi@sund.ku.dk

Running title: Landscape of *aph(3′)-Ia* in *E. coli* from livestock

**Supplementary methods**

*Isolate collection from Denmark*

The Danish genome collection comprised 120 *E. coli* clinical isolates obtained between 1992 and 2023. Of these, 113 originated from swine (94.2%), and were investigated in a previous study,^1^ while the remaining isolates originated from cattle (6; 5.0%) and a single dog (0.8%). DNA from bovine and canine isolates was extracted using the Maxwell RSC Cultured Cells DNA Kit (Promega, Wisconsin, USA) and sequenced on MiSeq (Illumina, California, USA) following the Nextera XT Library Preparation Kit (Illumina). *De novo* generated Illumina raw sequencing reads were assembled using SPAdes.^2^ Short-read sequencing was performed for all strains, while 7 porcine isolates (10.7%) were additionally long-read sequenced on the MinION platform (ONT, United Kingdom).

*Isolate collection from France*

The French genome collection comprised 152 *E. coli* clinical isolates obtained between 2006 and 2024. The isolates originated from cattle (101; 66.4%), swine (45, 29.6%) and dogs (6; 4.0%). DNA was extracted using the NucleoSpin microbial DNA mini kit (Macherey-Nagel, Hoerdt, France) and sequencing (Illumina NovaSeq 6000) was outsourced to Eurofins Genomics (Germany). Short-read sequencing was performed for all strains, while 4 isolates (two from cattle, two from swine) were additionally long-read sequenced using the MinION technology (ONT, United Kingdom). *De novo* generated raw sequencing reads were assembled using Unicycler.^3^

*Isolate collection from Netherlands*

The Dutch isolate collection comprised 316 neomycin resistant *E. coli* isolates obtained between 2021 and 2024. Amon these, 177 were obtained from caecal/faecal samples from the monitoring program for antimicrobial resistance in 2024 conducted within the framework of the EU Harmonized Monitoring Programme on AMR in accordance with EU Implementing Decision 2020/1729. Selective isolation was performed on MacConkey agar plates with 16 mg/L neomycin. The prevalence in veal calves was 57.5% (n=69 out of 120 samples), in broilers 52.3% (n=60 out of 108 samples), in pigs 38.7% (n=48 out of 124 samples), and in dairy cattle 1% (n=1 out of 113 samples). Long-read sequencing analysis was performed for all obtained isolates (n=177) using the Promethion 2 solo (ONT, United Kingdom). *De novo* raw sequencing reads were assembled using Hybracter.^4^

In addition, available genome data of 136 selectively isolated ESBL/AmpC-producing *E. coli* and 3 commensal *E. coli* isolated in 2021-2023, which were either shown to contain the neomycin resistance genes, were added to the study. These comprise of 16 isolates from broilers, 23 isolates form dairy cattle, 88 isolates from veal and 12 isolates from pigs. These 139 isolates were sequenced with Illumina short reads as previously described.^5^

*Isolate collection from United Kingdom*

The United Kingdom collection comprised 98 *E. coli* isolates obtained between 2013 and 2020. These originated from pigs (n=62, 63.3%), broilers (n=23, 23.5%), cattle (n=11, 11.2%), and turkeys (n=2, 2.0%). Of these, 36 isolates collected in 2017 (24 from pigs, 10 from cattle, and 2 from broilers) had been investigated previously,^6^ with 33 sequenced using a combination of short- and long-read technologies. The remaining 62 isolates were short-read sequenced as part of the EU Harmonized Monitoring Program on AMR conducted under EU Decision 652/2013. Raw sequencing reads were assembled using Unicycler.^3^

*Isolate collection from Sweden*

The Swedish genome collection comprised 64 *E. coli* isolates obtained between 2000 and 2024. The isolates originated from pigs (22; 34.4%), cattle (11, 17.2%), broilers (10; 15.6%), dogs (10; 15.6%), horses (9, 14.1%) and cats (2; 3.1%). DNA was extracted from overnight cultures on horse blood agar using EZ1 DNA tissue kit (Qiagen, Halden, Germany), according to the recommendations of the manufacturer. DNA was sent to Clinical Genomics Stockholm, Science for Life Laboratory (Solna, Sweden) for library preparation and short-read sequencing using Illumina technologies. Reads were assembled with SKESA.^7^

**References**

1. Subramani P, Menichincheri G, Pirolo M, *et al*. Genetic background of neomycin resistance in clinical *Escherichia coli* isolated from Danish pig farms. *Appl Environ Microbiol* 2023; **89**: e0055923.

2. Bankevich A, Nurk S, Antipov D, *et al*. SPAdes: A new genome assembly algorithm and its applications to single-cell sequencing. *J Comput Biol* 2012; **19**: 455–77.

3. Wick RR, Judd LM, Gorrie CL, Holt KE. Unicycler: Resolving bacterial genome assemblies from short and long sequencing reads. *PLOS Computat Biol* 2017; **13**: e1005595.

4. Bouras G, Houtak G, Wick RR, *et al*. Hybracter: enabling scalable, automated, complete and accurate bacterial genome assemblies. *Microb Genom* 2024; **10**: 001244.

5. Brouwer MSM, Zandbergen Van Essen A, Kant A, *et al*. Implementation of WGS analysis of ESBL-producing *Escherichia coli* within EU AMR monitoring in livestock and meat. *J Antimicrob Chemother* 2023; **78**: 1701–4.

6. AbuOun M, Jones H, Stubberfield E, *et al*. A genomic epidemiological study shows that prevalence of antimicrobial resistance in *Enterobacterales* is associated with the livestock host, as well as antimicrobial usage. *Microb Genom* 2021; **7**: 000630.

7. Souvorov A, Agarwala R, Lipman DJ. SKESA: strategic k-mer extension for scrupulous assemblies. *Genome Biol* 2018; **19**: 153.

**Supplementary figures**


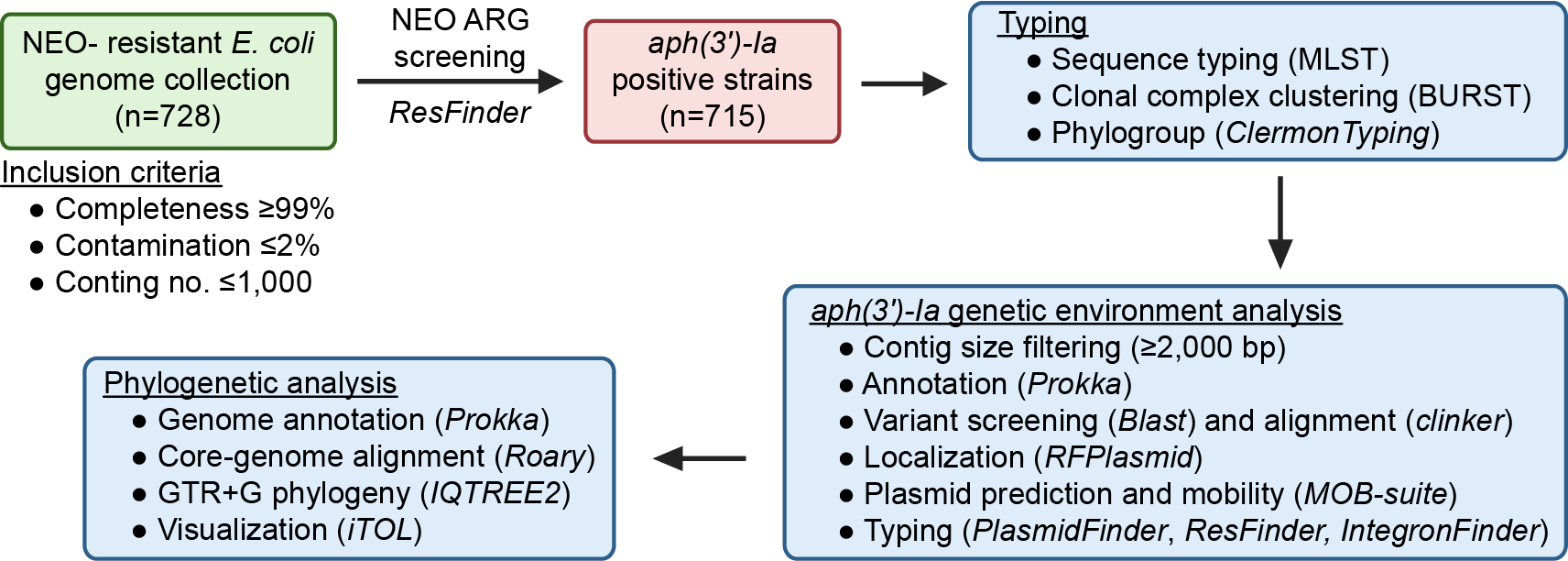


**Figure S1.** Schematic overview of the genetic analysis pipeline.


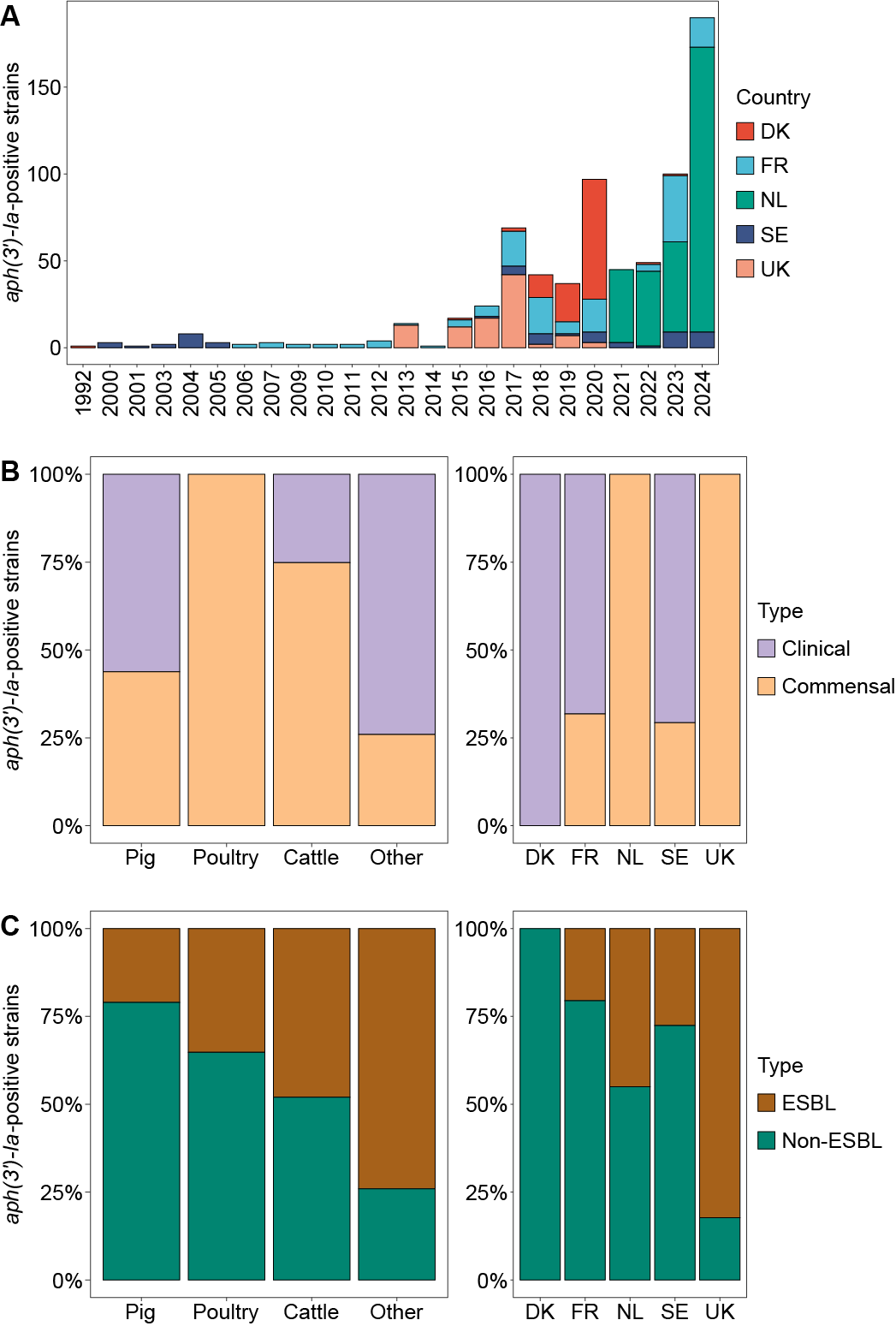


**Figure S2.** Collection year, clinical origin, and ESBL status of *aph(3’)-Ia*–positive *E. coli* isolates. A) Distribution of strain collection years, stratified by country of origin. B) Proportions of clinical and commensal strains in each host and country. C) Proportions of ESBL-producing and non-producing strains in each host and country.


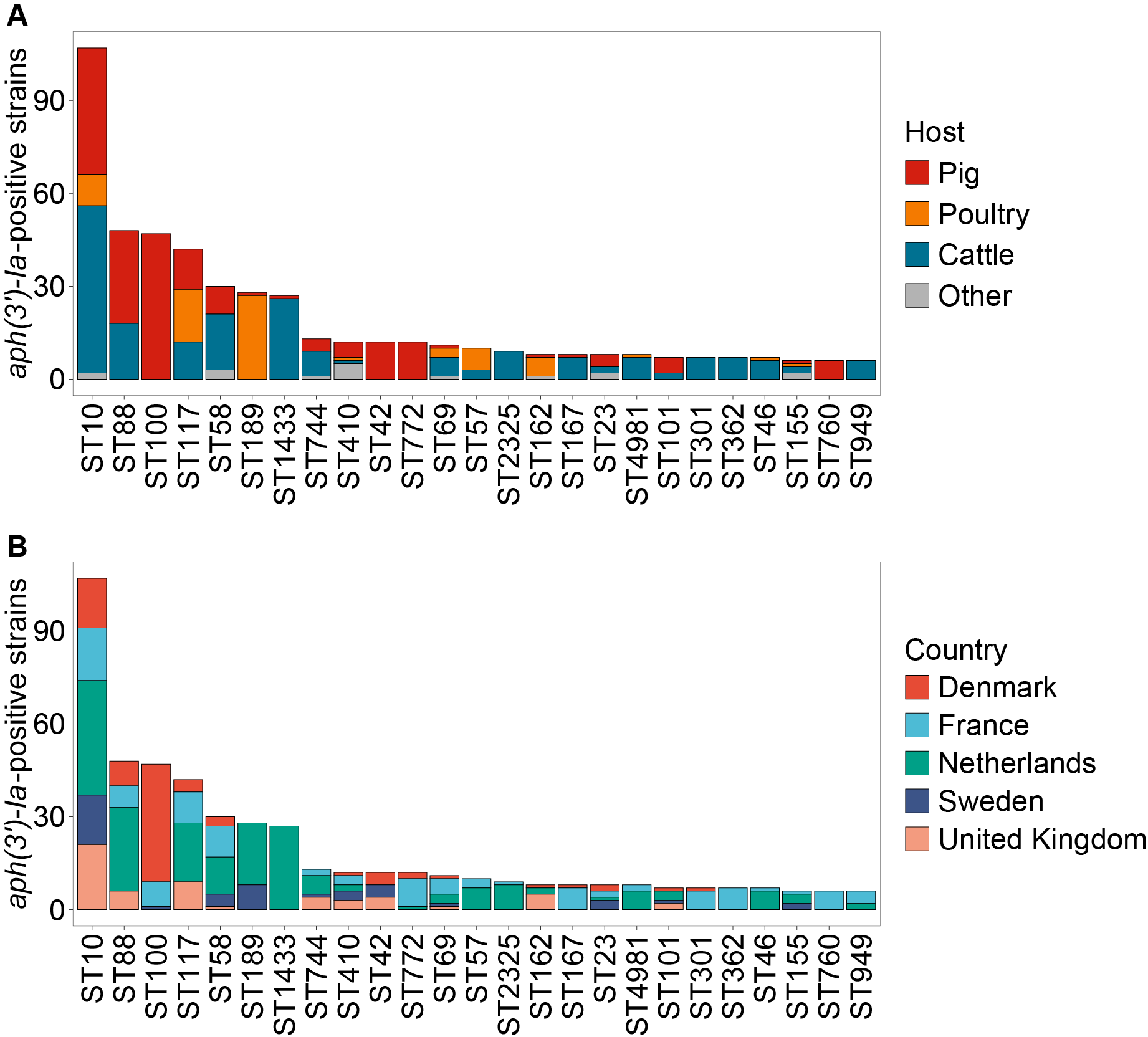


**Figure S3.** Distribution of the main sequence type (ST) among different hosts (A) and countries (B). Only STs with >5 isolates are displayed.


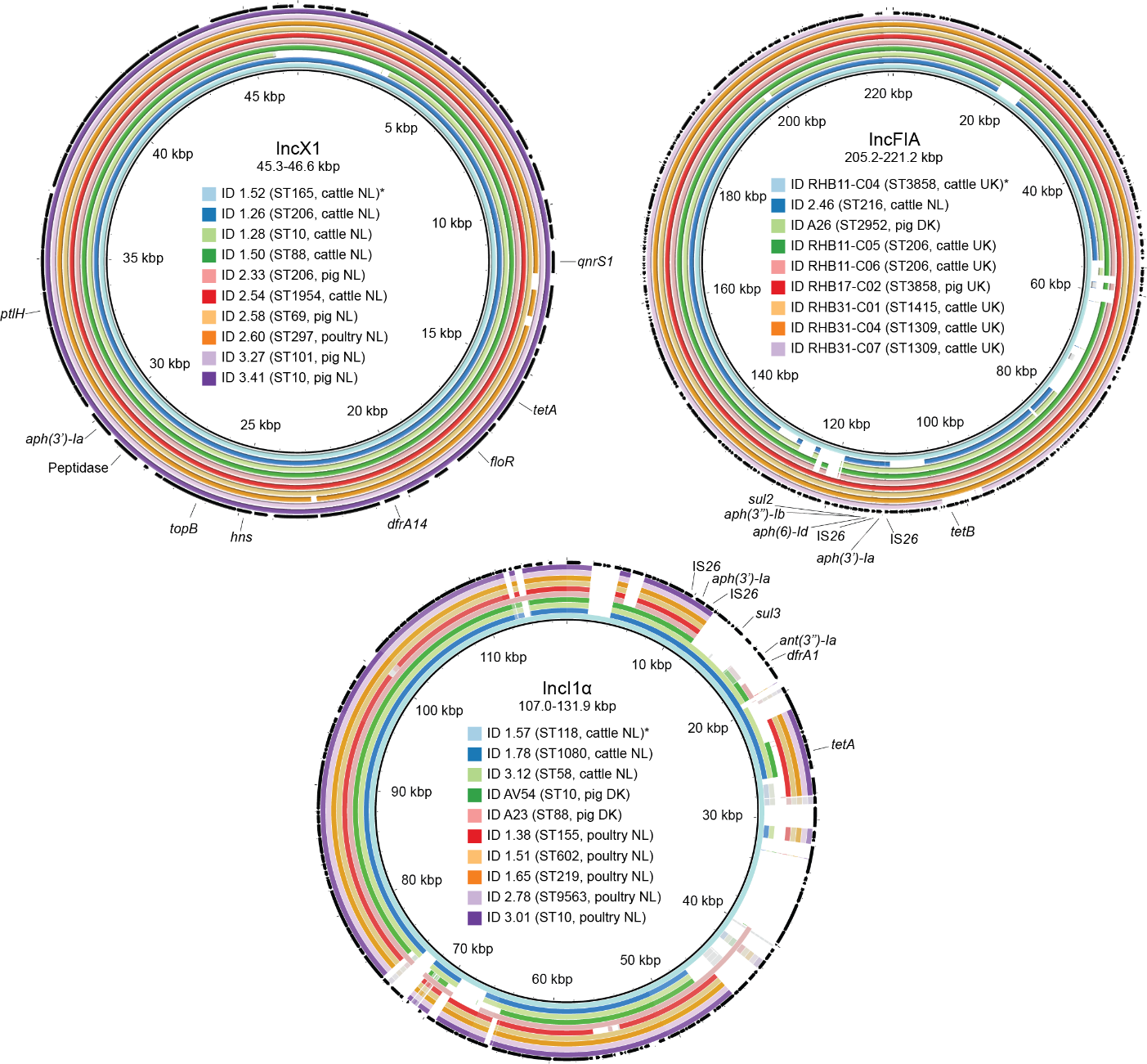


**Figure S4.** Comparative genomic maps of the predominant *aph(3′)-Ia*-carrying plasmid replicon types, namely IncX1 (top left), IncFIA (top right), and IncI1α (bottom). Each ring represents an individual *E. coli* isolate sequenced using long reads, selected to capture diversity across hosts, countries, and sequence types (STs). Asterisks (*) indicate reference plasmids used for alignment. The outer track depicts the *aph(3′)-Ia* genetic environment and co-located antimicrobial resistance genes.


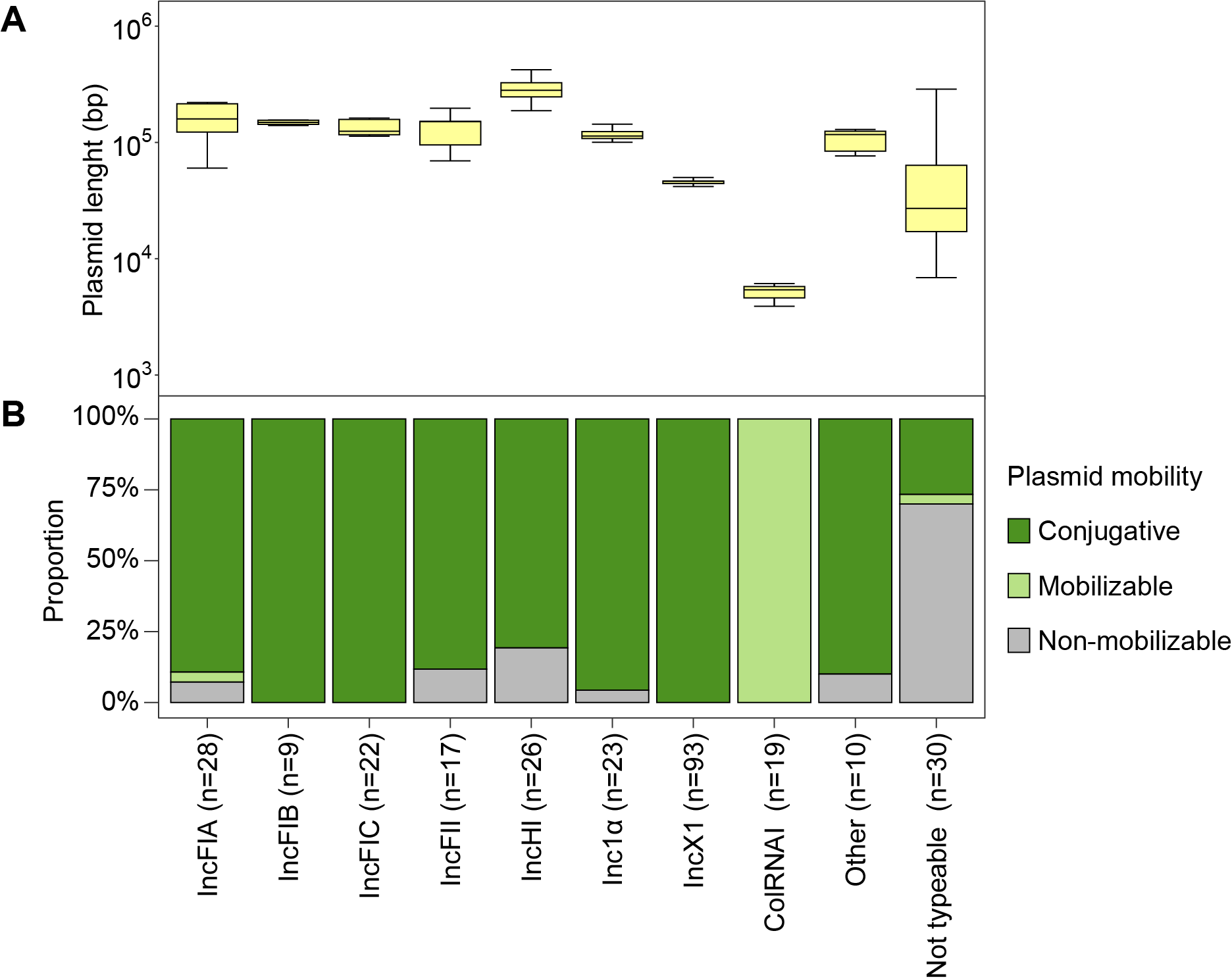


**Figure S5.** Distribution of plasmid length (panel A) and MOB-suite plasmid mobility prediction (panel B).


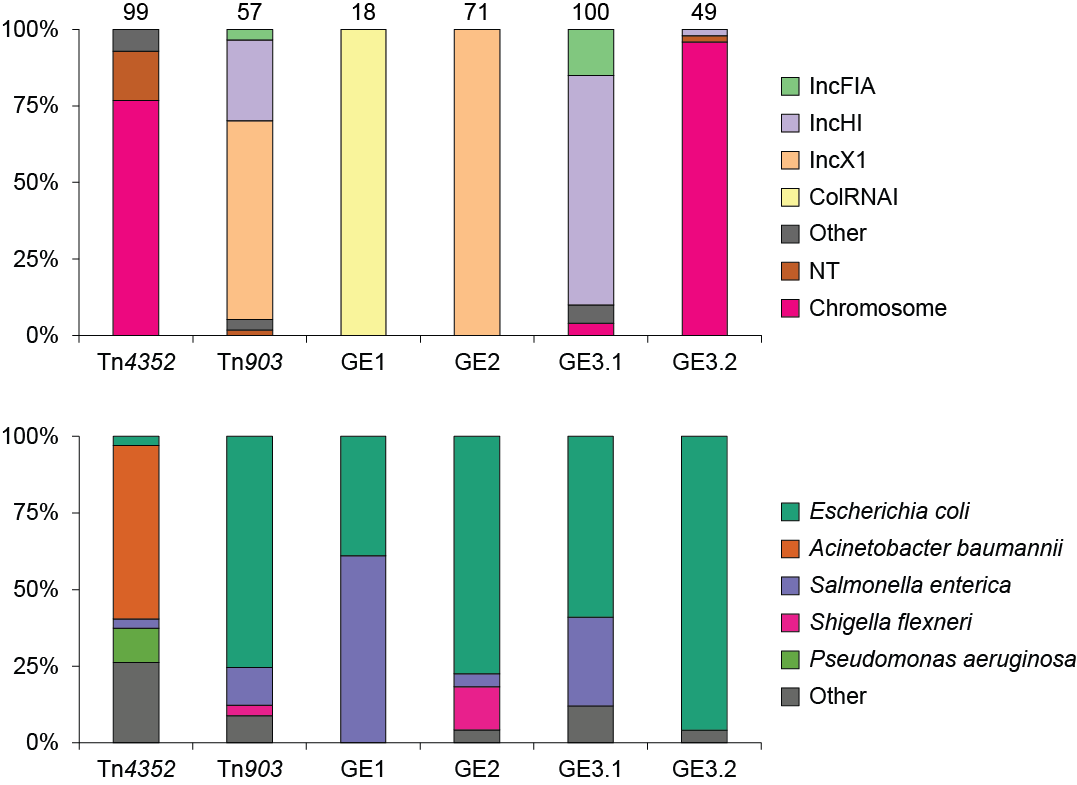


**Figure S6.** Distribution of *aph(3′)-Ia* genetic elements (GE) by plasmid replicon type or chromosomal sequences (top) and bacterial host species (bottom) based on BLASTn search of representative sequences (top 100, hits ≥95% identity and coverage). Numbers above bars indicate the total number of matches retrieved for each GE.


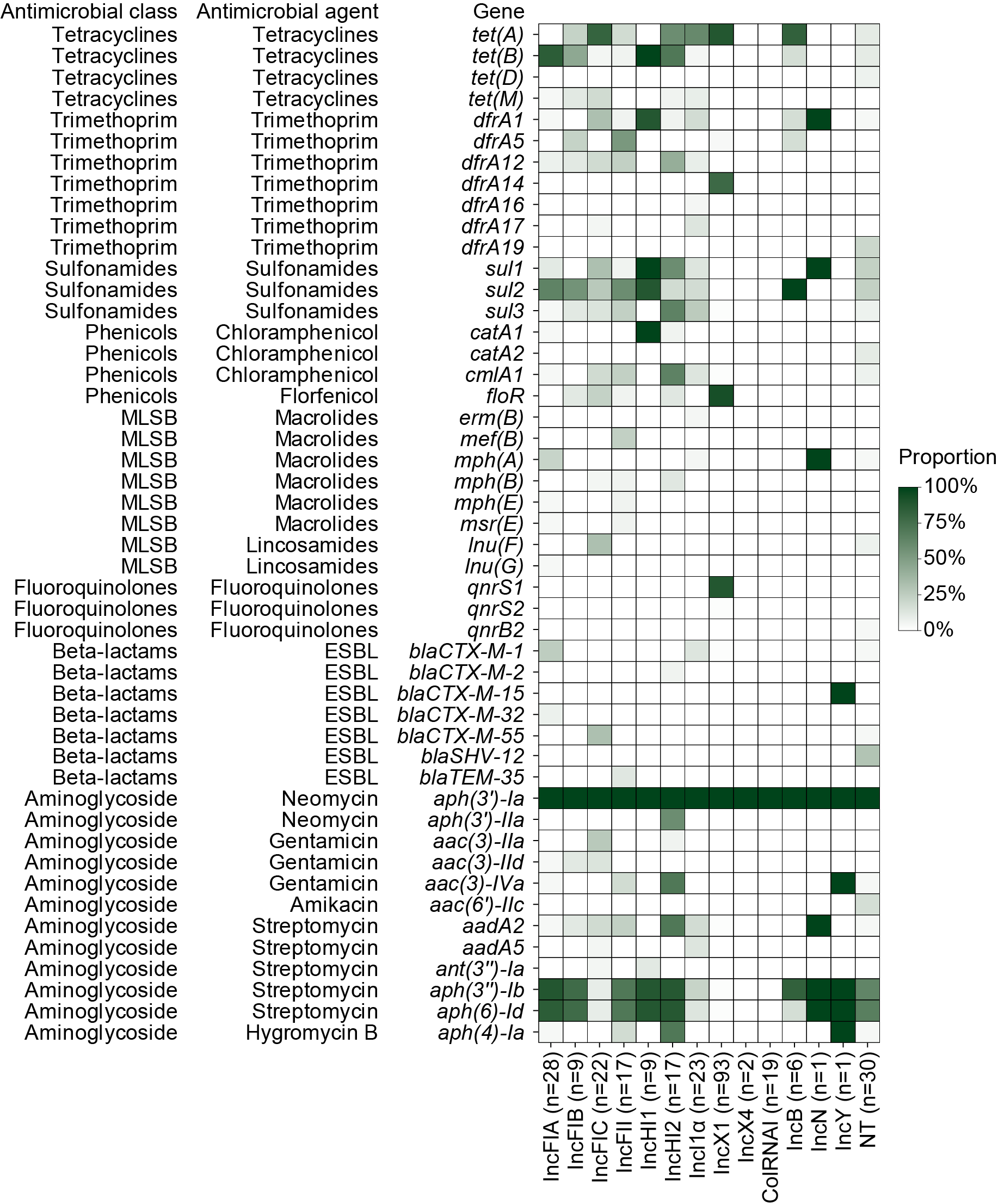


**Figure S7.** Distribution of antimicrobial resistance genes across plasmid replicon types. Abbreviations: ESBL, extended spectrum beta-lactamase; MLSB, macrolide-lincosamide-streptogramin B; NT, not-typeable plasmid.
